# Using Temporally and Spatially Resolved Measurements to Improve the Sensitivity of Fluorescence-Based Immunoassays

**DOI:** 10.3390/bios14050220

**Published:** 2024-04-28

**Authors:** Ran Kremer, Shira Roth, Avital Bross, Amos Danielli, Yair Noam

**Affiliations:** Faculty of Engineering, The Institute of Nanotechnology and Advanced Materials, Bar-Ilan University, Max and Anna Webb Street, Ramat Gan 5290002, Israelshirakoren111@gmail.com (S.R.);

**Keywords:** immunoassays, magnetic beads, image processing, signal processing, in vitro diagnostics

## Abstract

Detecting low concentrations of biomarkers is essential in clinical laboratories. To improve analytical sensitivity, especially in identifying fluorescently labeled molecules, typical optical detection systems, consisting of a photodetector or camera, utilize time-resolved measurements. Taking a different approach, magnetic modulation biosensing (MMB) is a novel technology that combines fluorescently labeled probes and magnetic particles to create a sandwich assay with the target molecules. By concentrating the target molecules and then using time-resolved measurements, MMB provides the rapid and highly sensitive detection of various biomarkers. Here, we propose a novel signal-processing algorithm that enhances the detection and estimation of target molecules at low concentrations. By incorporating both temporally and spatially resolved measurements using human interleukin-8 as a target molecule, we show that the new algorithm provides a 2–4-fold improvement in the limit of detection and an ~25% gain in quantitative resolution.

## 1. Introduction

Detecting target molecules, such as proteins, antibodies, or specific DNA sequences, within a population of molecules is critical in medical laboratory science. A typical molecule detection system consists of three elements: (a) a biological recognition component that captures the target molecule, (b) a reporting element, such as a fluorescent dye, quantum dots, or gold nanoparticles, that translates the biorecognition event into an analytically valuable signal, and (c) a detector that captures the physical signal [1]. Due to their high sensitivity [2,3,4] and multiplexing capabilities [5], optical sensing techniques [2,3,5,6] form the backbone of clinical diagnostic devices. In a typical fluorescence-based assay, the biological recognition element is immobilized onto a capture surface, such as a 96-well plate [7] or magnetic beads [8,9,10]. The target molecule is tagged either by a fluorescent molecule or by an enzyme to which a fluorescent substrate is added [11].

Optical detection systems usually consist of a photodetector [2,12,13,14], such as a photomultiplier tube (PMT) or an avalanche photodiode (APD), or a camera [4,15,16], e.g., a charged coupled device (CCD) or a complementary metal-oxide semiconductor (CMOS). A camera image provides two-dimensional spatial information but is less effective when the signal of the physical phenomenon is weak and embedded within background noise. A photodetector enables time-resolved measurements that increase optical detection sensitivity, particularly in detecting and identifying single fluorescent molecules. For example, confocal fluorescent microscopy with a photodetector can detect the emission from a single fluorescent molecule when it traverses a picoliter detection volume. This small detection volume significantly reduces background noise from the spurious fluorescence of impurities and Raman scattering of solvent molecules [17,18,19].

Time-resolved detection methods include fluorescence fluctuation spectroscopy (e.g., fluorescence correlation spectroscopy and fluorescence cross-correlation spectroscopy), single-molecule fluorescence burst detection [12], and pulse excitation with time-gated electronics [14]. While these methods are sensitive, they require expensive and sophisticated optics, and thus are less applicable in point-of-care applications. Moreover, they cannot capture the spatial information that a camera image provides. Camera-based detection is used in several clinical and research devices, such as the MagPix™ (Luminex Co., Austin, TX, USA) [15,20] and SIMOA™ (Quanterix Co., Billerica, MA, USA) [4]. However, these devices receive 2D spatial data from stationary target molecules. Therefore, data analysis relies only on signal intensity rather than spatial characteristics, such as the spatial distribution of light, the number of bead aggregates, and their size.

**Figure 1:**
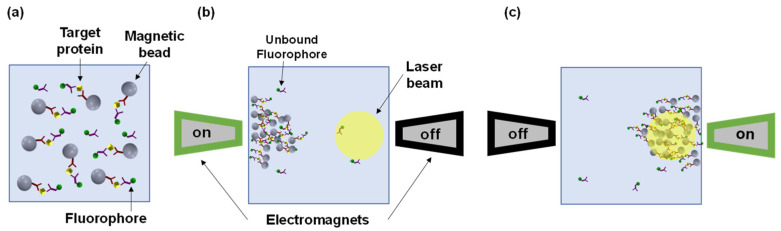
Illustration of the magnetic modulation biosensing (MMB) operation modes. (**a**) Target molecules are captured using magnetic beads with captured antibodies and tagged by fluorescently labeled detection antibodies. (**b**) The magnetic beads are aggregated and moved towards an electromagnet, while the unbound fluorescently labeled antibodies remain dispersed in the sample cell. When the left electromagnet is active, the magnetic beads are aggregated and move towards the left side of the sample cell, away from the laser beam, which is focused on the right side of the sample holder. During that time, the emitted background signal is recorded. (**c**) When the right electromagnet is active, the cluster of magnetic beads moves towards the right side of the sample holder, entering the laser beam, and the emitted fluorescence signal of the fluorescently labeled antibodies is recorded.

Recently, we presented a novel technology, termed magnetic modulation biosensing (MMB), and demonstrated its analytical and clinical sensitivity in various serological [21,22] and molecular assays [23], including assays for Zika and SARS-CoV-2. In an MMB-based assay, magnetic beads and fluorescently labeled probes are attached to the target analyte to form a “sandwich” (Figure 1a) [24]. Each magnetic bead (e.g., 6.5 µm in diameter) is conjugated to millions of capture antibodies, and therefore at different concentrations of target molecules, the number of fluorescently labeled antibodies per bead varies between zero (no target molecules in the solution) and millions (a high concentration of target molecules). An alternating external magnetic field gradient condenses the magnetic beads (and thus the target molecules with the fluorescently labeled probes) into the detection volume. The alternating field gradient sets them in periodic motion in and out of the laser beam, and thereby enables the removal of the constant background signal from the oscillating target signal without complicated sample preparation. Thus, MMB-assisted assays are characterized by both high sensitivity and shorter and less work-intensive testing protocols [22]. For example, using the MMB system, we detected human interleukin 8 (IL-8) within 15 min and demonstrated a limit of detection (LoD) of 0.08 ng/L and a 4-log dynamic range, much better than the state-of-the-art ELISA (1.5 ng/L LoD and 2-log dynamic range) [24]. Moreover, using the MMB system and the Zika virus NS1 protein (as a capture antigen) to detect anti-NS1 Zika IgG and IgM antibodies, we blindly tested 60 reverse transcription-PCR Zika-positive samples and healthy patients’ serum samples. The MMB-based Zika assays had 100% specificity and 88–97% sensitivity, significantly higher than the state-of-the-art Euroimmun ELISA assays (38–74%) [21].

The initial version of the MMB system detected the oscillating fluorescence signal with a photomultiplier and demodulated it with a lock-in amplifier [24]. Unlike other optical detection systems that measure the signal from individual beads, the MMB system collects the fluorescence signal from multiple beads, resulting in a relatively strong signal. With such an intense signal, a simple camera, rather than an expensive PMT, can analyze the intensity of oscillating fluorescent molecules. Consequently, in more recent versions of the MMB system, the PMT was replaced by a camera [23]. The inexpensive camera also offers other key advantages, such as 2D information.

**Figure 2:**
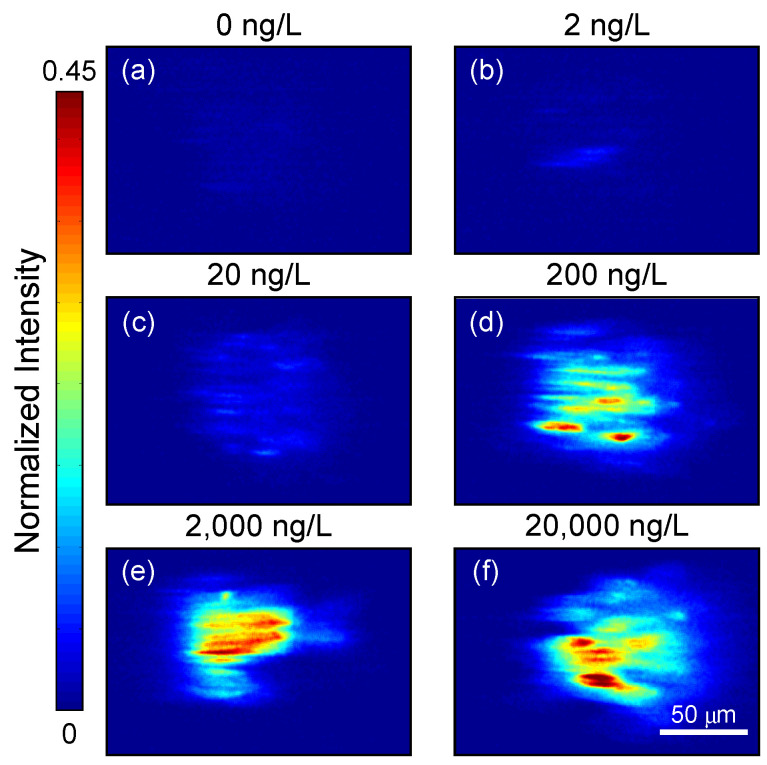
Snapshots of magnetic beads passing through a laser beam in various concentrations of a target protein, interleukin-8 (IL-8), in a buffer solution. (**a**) 0 ng/L, (**b**) 2 ng/L, (**c**) 20 ng/L, (**d**) 200 ng/L, (**e**) 20,000 ng/L, and (**f**) 200,000 ng/L. In each experiment—conducted with a specific IL-8 concentration—the camera collected a total of 600 frames over a 12-s period. The size of each frame is rectangular (300 × 300 pixels), and the 50 µm bar indicates the actual size for both the x- and y-axes.

As an example, Figure 2 shows snapshots of magnetic beads passing through the laser beam in various concentrations of IL-8 in a buffer solution. When the concentration of the IL-8 in solution is high, we can see aggregates of fluorescently labeled target molecules inside the laser beam. However, at intermediate and low concentrations, we can see dotted and blurred aggregates, respectively (Figure 2). At each modulation cycle, the beads traverse the area illuminated by the laser beam, thus inducing a different spatial signature. When the beads aggregate entirely inside the laser beam (“on-frame”), the image is brighter than when the aggregate is entirely outside the laser beam (“off-frame”). The resulting signal is periodic at the modulation frequency of the electromagnets (1 Hz), and each video frame contains spatial information. For example, the “on-frames” have different spatial structures depending on the target molecule concentration.

While the MMB system provides the highly sensitive detection of various target molecules, it currently relies solely on time-resolved measurements [21,22,23,24]. Despite the 2D information collected by the camera, the signal processing algorithm ignores the spatially resolved information and simply sums up all the fluorescent signals captured by the camera, similar to a photodetector. Here, to improve the sensitivity of the MMB system, we design signal processing algorithms that utilize both temporally and spatially resolved information. To evaluate the new algorithms, we use the MMB-based IL-8 assay and conduct four experiments. The results demonstrate that the new algorithms offer a 2–4-fold improvement in the limit of detection (LoD), while maintaining or enhancing quantitative resolution (QR).

## 2. Materials and Methods

### 2.1. Magnetic Modulation Biosensing (MMB) System Description

The camera-based MMB system [23] uses a 532 mm laser diode (CPS532, Thorlabs, Newton, NJ, USA) working at 0.25 mW that generates a 3.5 mm diameter beam. The laser beam passes through a pair of plano-convex lenses (200 mm and 50 mm focal lengths, Thorlabs), a dichroic beam splitter (Di02-R532, Semrock, Rochester, NY, USA), and finally an objective lens (M-10X, 0.25NA, Newport, Irvine, CA, USA) that focuses the beam to a 150 µm diameter on a rectangular sample cell (W2540, Vitrocom, Mountain Lakes, NJ, USA) containing the fluorescently labeled probes, target molecules, and magnetic beads. Two electromagnets generate an alternating magnetic field gradient at 1 Hz, which pulls the magnetic beads and concentrates them into a small detection area, thus increasing the fluorescence-detection sensitivity. The oscillating magnetic-field gradient drives the aggregated beads from side to side, in and out of the laser beam (Figure 1), thereby enhancing the signal emitted from the fluorescence molecules bound to the beads compared to the background noise, which is induced by Raman scattering from the solvent molecules or unbound fluorescent molecules. The emitted fluorescence is collected by the same objective lens, filtered by two emission filters (Semrock, FF03-575/25-25), and detected by a digital camera (FLIR, GS3-U3-23S6M-C). For each experiment, within 12 s, the camera acquires 600 images at 50 frames per second.

### 2.2. Interleukin-8 Assay

For our MMB-based interleuikin-8 (IL-8) assay, we used the commercially available Bio-Plex Pro Human Chemokine IL-8 assay (CXCL8 Set #171BK31MR2, BioRad, Hercules, CA, USA). To reduce the background autofluorescence of the magnetic beads prior to using them in the assay, we photobleached them for two hours [25]. In each well of a 96-well plate, we mixed 50 μL of X2 photobleached conjugated magnetic beads with 50 μL of solution at one of eight different concentrations of a recombinant human IL-8 protein (#574202, BioLegend, San Diego, CA, USA). The overall concentration levels were 0.05, 0.1, 0.2, 1, 2, 5, 20, and 200 ng/L. After one hour of incubation, we added 50 μL of detection antibodies (X1) to each well and further incubated it for 30 min. We then added 80 μL of X1 streptavidin phycoerythrin SA-PE (Biso-Plex Pro Reagent Kit III #171304090M, BioRad, Hercules, CA, USA) and incubated it for another 20 min. All incubations were performed at room temperature on a rotator. Finally, we washed the magnetic beads once by placing the 96-well plate on a MagJET separation rack (MR02, Thermo Fisher Scientific, Waltham, MA, USA) for four minutes, removing the solution, and pipetting the beads with 200 μL of an assay buffer (PBS X1, 1% BSA *w*/*v*, 0.05% Tween-20). The washed beads were placed again on the separation rack, and the buffer was replaced with 100 μL of assay buffer for MMB measurements.

To collect sufficient data for statistical analysis [26], we conducted four experiments on four different days. Each experiment included eight independent blank samples and four independent samples for each IL-8 concentration.

### 2.3. Mathematical Model

For each experiment, the MMB system generated a twelve-second video at 50 frames-per second (fps). Each video is denoted by ${\left\{Z\left(n\right)\right\}}_{n=1}^{N}$, where N=600 is the total number of frames per experiment. Each $Z(n)\in R^{M\times M}$ is a matrix representing a single video frame (image) at time instance n, where M=1024 is the number of pixels in each row and column. For each frame, ${\left[Z\left(n\right)\right]}_{i,j}$ represents the (i,j) entry, which corresponds to the light intensity impinging upon the corresponding camera pixel at location (i,j). Mathematically, for a given target molecule concentration c, each frame satisfies the following equation:

(1) $${\left[Z\left(n\right)\right]}_{i,j}=kb\left(i,j,i_{0},j_{0}\right)s\left(i,j,n,c\right)+v\left(i,j,n\right)$$

where s(i,j,n,c) indicates the fluorescent molecule concentration at a point in the sample holder that is imaged to pixel (i,j) at time instance n, $b(i,j,i_{0},j_{0})$ denotes the normalized laser spatial-signature centered at position $\left(i_{0},j_{0}\right)$, k is a constant proportional to the laser intensity, and v(i,j,n) denotes the spatiotemporal noise, which is contributed by Raman scattering of the water molecules, unbound fluorescent molecules, the autofluorescence emitted from the beads [25], and shot noise.

### 2.4. Signal Processing Algorithms

To test whether a combination of spatially and temporally resolved analyses of the data acquired by the camera can improve the analytical performance of the MMB system, we applied two algorithms, namely “Feature 1” and “Feature 2”, and compared their analytical performance (LoD and QR) with the results of the time-resolved analysis, the “Baseline approach”. Each algorithm included a preliminary process followed by a feature extraction phase. The preliminary process detected the laser beam position, generated a pinhole mask, and reduced the size of each frame to focus on the region of interest centered at the beam. Then, we used spatial processing to transform ${\left\{Z\left(n\right)\right\}}_{n=1}^{N}$ into a one-dimensional time-series y(n), dubbed the temporal feature. Finally, we applied time-domain signal processing to extract the scalar feature x that constitutes the response.

#### 2.4.1. Preliminary Process

First, we detected the laser-beam center by averaging the video frames $\bar{Z}=\frac{1}{N}\sum_{n=1}^{N}Z\left(n\right)$ and calculating the $\bar{Z}$ center of mass ($z_{x},\;z_{y}$), using

(2) $$z_{x}=\frac{\sum_{i=1}^{M}{i\left[z_{x}\right]}_{i}}{\sum_{i=1}^{M}{\left[z_{x}\right]}_{i}}\;\mathrm{and}\;z_{y}=\frac{\sum_{j=1}^{M}{j\left[z_{y}\right]}_{j}}{\sum_{j=1}^{M}{\left[z_{y}\right]}_{j}},$$

where ${\left[z_{x}\right]}_{i}=\sum_{j=1}^{M}{\left[\bar{Z}\right]}_{i,j}$ and ${\left[z_{y}\right]}_{j}=\sum_{i=1}^{M}{\left[\bar{Z}\right]}_{i,j}$.

Second, we defined a new matrix $P\in R^{M\times M}$, which is a circular pinhole mask with a radius of 150 pixels around the laser beam center, transferring only the region of interest:

(3) $${\left[P\right]}_{i,j}=\left\{\begin{array}{cc} 1 & {\left(i-z_{x}\right)}^{2}+{\left(j-z_{y}\right)}^{2}\leq 150^{2}\\ 0 & otherwise \end{array}\right..$$

Then, for each frame $Z\left(n\right)$, we performed $P\circ Z\left(n\right)$, where (∘) is the Hadamard product (the entry-wise product of the two matrices).

Third, to reduce computational complexity, we cropped the size of each frame by extracting from each $Z\left(n\right)$ frame a 300 × 300 frame $\tilde{Z}\left(n\right)$ centered at ($z_{x},\;z_{y}$):

(4) $${\left[\tilde{Z}\left(n\right)\right]}_{i,j}={\left[Z\left(n\right)\right]}_{z_{x}-150+i,z_{y}-150+j},\;0\leq i,j\leq 300.$$

Henceforth, for brevity, we will refer to $\tilde{Z}\left(n\right)$ as $Z\left(n\right)$.

#### 2.4.2. Baseline Approach

For the baseline approach, to emulate the photomultiplier tube (PMT), we formed a one-dimensional scalar time-series by mapping each video frame ${\left\{Z(n)\right\}}_{n=1}^{N}$ to its squared Frobenius norm:

(5) $${\tilde{y}}_{BL}(n)=\sum_{i=1}^{300}\sum_{j=1}^{300}{\left[Z\left(n\right)\right]}_{i,j}^{2}.$$

To smooth the one-dimensional time-series, we applied a 12-length median filter:

(6) $$y_{BL}\left(n\right)=\mathrm{median}\left\{{\tilde{y}}_{BL}\left(\max\left\{n-5,0\right\}\right),\ldots ,{\tilde{y}}_{BL}\left(\min\left\{n+6,N\right\}\right)\right\}.$$

Then, we applied a 4 Hz cutoff lowpass filter. The final output was the resulting signal energy, denoted by $x_{BL}$:

(7) $$x_{BL}=\sum_{n=1}^{N}y_{BL}^{2}\left(n\right).$$

#### 2.4.3. Feature 1

In Feature 1, to remove outliers in each frame $Z\left(n\right)$, we trimmed the frames and kept only the pixels corresponding to the reference interval within the 80th to 99.5th percentiles. Let $\alpha_{U}$ and $\alpha_{L}$ be the 99.5th and 80th percentiles, respectively, and then

(8) $${\left[Z_{1}\left(n\right)\right]}_{i,j}=\left\{\begin{array}{cc} {\left[Z\left(n\right)\right]}_{i,j} & \alpha_{L}\leq {\left[Z\left(n\right)\right]}_{i,j}\leq \alpha_{U}\\ 0 & otherwise. \end{array}\right.$$

Then, we reduced the resulting video ${\left\{Z_{1}(n)\right\}}_{n=1}^{N}$ to a one-dimensional time-series ${\tilde{y}}_{1}(n)$ of length N, where each ${\tilde{y}}_{1}(n)$ is the first singular value in the singular value decomposition (SVD) of $Z_{1}(n)$. Explicitly, the SVD of $Z_{1}(n)$ is

(9) $$(n)\Sigma (n)W^{†}(n)=Z_{1}(n),$$

where $U\left(n\right),W(n)\in C^{L\times L}$ are unitary matrices (with L=300) and $\Sigma (n)\in R^{L\times L}$ is a diagonal matrix containing the singular values $\sigma_{m}(n),m=1,\ldots ,L$ in decreasing order; that is, $\left[\Sigma (n){]}_{ij}=\delta_{i-j}\sigma_{i}(n\right)$ with $\sigma_{1}(n)\geq \sigma_{2}(n)\geq \cdots \sigma_{L}(n)\geq 0$, $\delta_{i}$ is the Kronecker delta. Therefore, the one-dimensional time-series of Feature 1 is

(10) $${\tilde{y}}_{1}(n)=\sigma_{1}(n).$$

To smooth the one-dimensional time-series, we applied the 12-length median filter:

(11) $$y_{1}\left(n\right)=\mathrm{median}\left\{{\tilde{y}}_{1}\left(\max\left\{n-5,0\right\}\right),\ldots ,{\tilde{y}}_{1}\left(\min\left\{n+6,N\right\}\right)\right\}.$$

Finally, we took the average energy of the *N*-length vector:

(12) $$x_{1}=\sum_{n=1}^{N}\;y_{1}^{2}(n).$$

#### 2.4.4. Feature 2

In Feature 2, we reduced the video ${\left\{Z(n)\right\}}_{n=1}^{N}$ (without trimming it first) into a one-dimensional time-series ${\tilde{y}}_{2}(n)$ of length N, where each ${\tilde{y}}_{2}(n)$ is the second singular value in the SVD of Z(n) (i.e., ${\tilde{y}}_{2}(n)=\sigma_{2}(n)$). Then, we used a time-domain filter with three equal passbands of Δf=0.5Hz, centered at 1, 3, and 5 Hz, respectively. When $h_{BF-3}(n)$ is the filter impulse response, the filter output is the linear convolution of ${\tilde{y}}_{2}(n)$ and the filter:

(13) $$y_{2}(n)=\left[{\tilde{y}}_{2}\;*\;h_{BP-3}\right](n).$$

Finally, we took the average energy of the *N*-length vector:

(14) $$x_{2}=\sum_{n=1}^{N}\;y_{2}^{2}(n)$$

#### 2.4.5. Homoscedasticity and Normality

To transform the responses to be normal and homoscedastic at low concentrations up to four times the limit of blank (LoB) [26,27], we used the natural logarithm function, which guarantees that the data are normal and homoscedastic [28]. We then tested the data for normality using Kolmogorov–Smirnov tests and tested for homoscedasticity using Levene’s and Conover’s tests. After corroborating normality and homoscedasticity, we used the Cedergreen–Ritz-–-Streibig model 24 to fit the dose–responses [29].

#### 2.4.6. Normalization

Because the average energy of each feature ranges over different intervals, we offset and normalized each feature using min–max normalization as follows: we let $x_{BL}^{c,i}$ be the baseline response at concentration c∈C for sample $i\in I_{c}$, where $I_{c}$ is the set of measurements taken at concentration c; then

(15) $$x_{BL}^{c,i}\leftarrow \left(x_{BL}^{c,i}-\frac{1}{\left|I_{0}\right|}\sum_{i=1}^{I_{0}}\;\;x_{BL}^{0,i}\right){\left(\frac{1}{\left|I_{c_{max}}\right|}\sum_{i=1}^{I_{c_{max}}}\;\;x_{BL}^{c_{max},i}\right)}^{-1}+1,\forall i,c,$$

where $c_{max}$ denotes the maximum concentration (200 ng/L), $I_{c_{max}}$ is the total number of measurements at $c_{max}$, and $I_{0}$ is the total number of measurements at the blank concentration. For example, we performed a total of 27 measurements at the blank concentration, and therefore at c=0 ng/L, the total number of measurements is $I_{0}=27$, and the index i=1…27. The same normalization was applied for Feature 1 and Feature 2 by replacing $x_{BL}^{c,i}$ in Equation (15) with $x_{1}^{c,i}$ and $x_{2}^{c,i}$, respectively.

#### 2.4.7. Dose–Response

After applying the natural logarithm function and using Equation (15) to normalize the data, we generated a dose–response and used the Cedergreen–Ritz–Streibig model [29] to fit the calibration function, $X\left(c\right)$. The quantitative resolution (QR) at each concentration is defined as the sample standard deviation divided by the calibration curve slope at that concentration.

#### 2.4.8. Levels of Blank and Detection

We determined the LoB and LoD via parametric tests [26,30,31]. For the LoB, we calculated the critical signal value [30] corresponding to α=5%, using

(16) $$S_{B}={\hat{\mu }}_{B}+1.645{\hat{\sigma }}_{B}^{2},$$

where ${\hat{\mu }}_{B}$ is the blank sample mean and ${\hat{\sigma }}_{B}^{2}$ is the blank sample variance. We then determined the LoB (in the concentration domain) from $S_{B}$ via the inverse dose–response (i.e., $LoB=X^{-1}\left(S_{B}\right)$).

For the LoD, the minimum detectable signal value is [26]

(17) $$S_{D}=S_{B}+c_{\beta }{\hat{\sigma }}_{p}.$$

Here, ${\hat{\sigma }}_{p}$ is the root pooled variance, $c_{\beta }$ is defined as

(18) $$c_{\beta }=\frac{1.645}{1-1/\left(4f_{L}\right)-3},$$

and $f_{L}$ is the number of degrees of freedom [30], which for 43 measurements at three different concentrations equals $f_{L}=43-3=40$. We estimated the root pooled variance at concentrations up to four times the LoB, which we had already confirmed to be homoscedastic. Explicitly, for Features 1 and 2, we used 0.05, 0.1, and 0.2 ng/L, whereas for the Baseline approach, we used 0.05, 0.1, 0.2, and 1 ng/L. Finally, we determined the LoD (in the concentration domain) from $S_{D}$ via the inverse dose–response (i.e., $LoD=X^{-1}\left(S_{D}\right)$).

#### 2.4.9. Detection Procedure

To estimate the concentration of a target molecule in the samples given a video ${\left\{Z\left(n\right)\right\}}_{n=1}^{600}$, where $Z\left(n\right)\in R^{1024x1024}$, the following steps should be taken:

1. Apply a preliminary process (Section 2.4.1) by finding the center of mass, define a pinhole mask, and crop each frame to create ${\left\{Z\left(n\right)\right\}}_{n=1}^{600}$, where $Z\left(n\right)\in R^{300\times 300}$
2. Use either the Baseline approach (Section 2.4.2), Feature 1 (Section 2.4.3), or Feature 2 (Section 2.4.4) algorithms to convert the series of frames to a one-dimensional scalar time-series ($y_{BL}\left(n\right)$, $y_{1}\left(n\right)$, or $y_{2}\left(n\right)$), and then convert it to a scalar ($x_{BL}$, $x_{1}$, or $x_{2}$).
3. Use the natural logarithm function (Section 2.4.5) and normalize the result (Section 2.4.6).
4. Determine the concentration using the inverse dose–response, $X^{-1}\left(x_{BL}\right)$, $X^{-1}\left(x_{1}\right)$, or $X^{-1}\left(x_{2}\right)$.

## 3. Results

**Figure 3:**
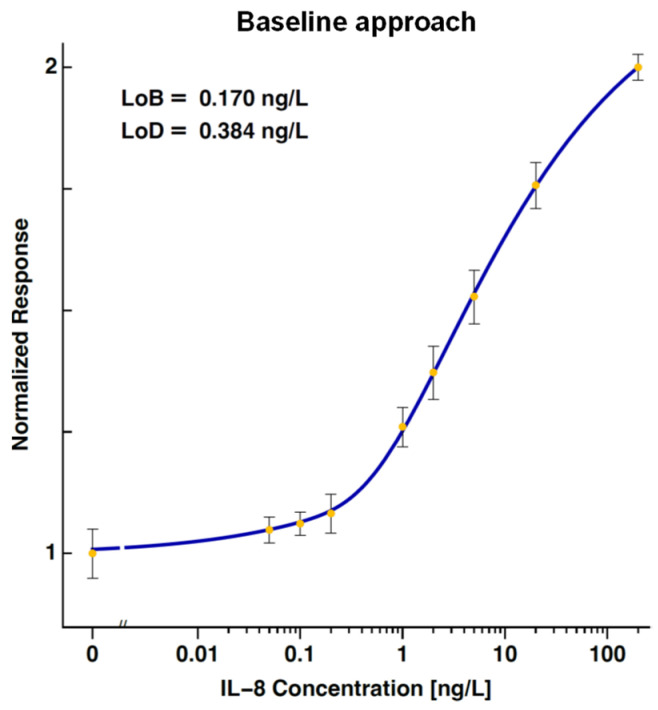
A dose–response curve for IL-8, established using the baseline approach. The calculated limit of blank and limit of detection are 0.170 ng/L and 0.384 ng/L, respectively. The error bars represent the standard deviations of the measurements at each concentration.

The dose–response curve of IL-8 established using the Baseline approach is shown in Figure 3. The calculated LoB and LoD were 0.170 ng/L and 0.384 ng/L. The concentrations of the IL-8 ranged between 0 and 100 ng/L, and the signal at the highest measured concentration was not saturated.

**Figure 4:**
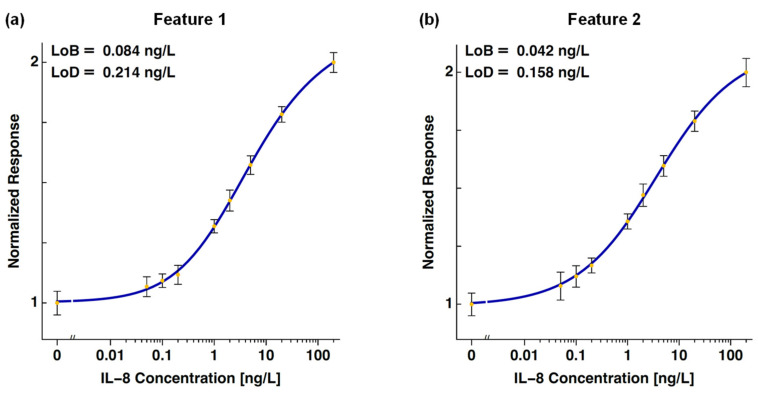
Dose–response curves of IL-8 established using the (**a**) Feature 1 and (**b**) Feature 2 algorithms. The calculated limits of blank are 0.084 ng/L and 0.042 ng/L, and the calculated limits of detection are 0.214 ng/L and 0.158 ng/L, respectively. The error bars represent the standard deviations of the measurements at each concentration.

The dose–response curves of IL-8 established using Feature 1 and Feature 2 are shown in Figure 4. The calculated LoB values are 0.084 ng/L and 0.042 ng/L, and the calculated LoD values are 0.214 ng/L and 0.158 ng/L, respectively.

To estimate the impact of Features 1 and 2 on the analytical performance of the MMB system, we summarized the LoB and LoD of each feature in Table 1. The gain was calculated as the ratio between the baseline LoD and the LoD of each feature. The percentage gain for each feature was calculated by subtracting the feature’s LoD from the baseline LoD and then dividing it by the baseline LoD. With the utilization of the new features, the gains in LoB and LoD ranged between 2 and 4.

The quantitative resolutions (QRs) at four concentrations (1, 2, 5, and 20 ng/L) for both the Baseline approach and Features 1–2 are summarized in Table 2. The percentage gain for each feature relative to the Baseline approach was calculated by subtracting the feature’s QR from the Baseline QR and then dividing it by the Baseline QR. The gain in QR for Feature 1 was ~25% at various concentrations. Feature 2 did not provide much gain in QR.

Table 3 presents the *p*-values resulting from the Kolmogorov–Smirnov tests for each method at five different concentrations (0, 0.05, 0.1, 0.2, and 1 ng/L). Because the *p*-values exceed the chosen significance level (0.05), there is no significant difference between the distributions of the measurements at various concentrations and the normal distribution. Therefore, the results support the conclusion that the data follow a normal distribution.

The *p*-values from Levene’s and Conover’s tests for each method are presented in Table 4. Because the *p*-values from both tests exceed the chosen significance level (0.05), there is no significant difference in variances among the different concentrations. Therefore, the results support homoscedasticity, and the calculations of the LoB and LoD are valid.

## 4. Discussion

Fluorescence-based immunoassays are widely employed in both research and clinical diagnostics. After a biorecognition event, a fluorescent signal is generated and detected using an optical detection system, typically comprising a photodetector or camera. Previously, we introduced a novel optical detection technology, termed MMB. To date, the MMB system has demonstrated rapid and high analytical sensitivity in multiple serological and molecular assays, such as for Zika [21] and SARS-CoV-2 [23]. A recent review of MMB technology extensively discussed its advantages (e.g., high analytical and clinical sensitivity, simple protocol, and relatively short time-to-result) as well as its limitations (e.g., low throughput, the requirement for a specific sample holder, and the use of relatively bulky electromagnets) [22].

Despite using a camera that acquires 2D information over time, the signal processing of the MMB system has thus far included only time-resolved measurements (i.e., the Baseline approach). In this study, we introduce two algorithms (“Feature 1” and “Feature 2”) that utilize both spatial- and time-resolved measurements and we compared their analytical performances with the Baseline approach. For statistical assessment, we conducted four experiments; each repeated the same procedure at the same concentration. Our results indicate that spatiotemporal processing significantly improves the limit of detection and the estimation error (i.e., the quantitative resolution).

Compared with the Baseline approach, Feature 1 improves the LoD and LoB by a factor of 1.8–2.1 and the quantitative resolution by approximately 25%. Feature 2 enhances the analytical performance even further, improving the LoD and LoB by a factor of 2.4–4.1. However, unlike Feature 1, Feature 2 does not improve the quantitative resolution when compared with the Baseline approach. These results can be explained by the spatial processing attributes associated with each feature. Unlike the Baseline approach, which emulates the PMT and takes all the energy in each frame, Feature 1 first performs a reference interval to remove outliers and then calculates the spectral norm (i.e., the first singular value). This allows Feature 1 to capture the most dominant spatial energy of each video frame, including the DC signal component. Additionally, it applies only a median filter in the time domain.

Because its spatial processing focuses solely on the second singular value, Feature 2 includes more subtle information. Although the second singular value contains less energy compared to the spectral norm (the first singular value), it remains informative and is not negligible relative to the noise. Additionally, the time domain signal processing of Feature 2 includes the triple band-pass filter, which further reduces noise. This filter eliminates the DC signal and reduces spectral components residing outside the filter’s narrow passbands at 1, 3, and 5 Hz. These passbands correspond to the first three harmonics of the Fourier series of the MMB modulating signal, which is a square wave at 1 Hz with presumably a 50% duty cycle. However, because the square wave is not pure—it deviates from a 50% duty cycle and is embedded in noise—the output signal contains information beyond these bands, including a DC component.

The calibration functions of the two features (Figure 4) include a linear interval. To increase the interval, another nonlinear transformation could be applied. However, such a transformation makes the data heteroscedastic, which drastically reduces the accuracy of the statistical performance analysis.

Due to the enhanced noise reduction, Feature 2 is more suitable for low-level detection than Feature 1. However, because of its improved QR, Feature 1 outperforms Feature 2 at higher concentrations. Hence, the noise reduction of Feature 2 at these concentrations is outweighed by its signal distortion. Future research may investigate utilizing Feature 2 as a classification algorithm to ascertain if the target concentration exceeds or falls below a specified threshold, such as the LoD. Once the concentration surpasses the LoD, Feature 1 can be leveraged to estimate the concentration with greater accuracy. Moreover, with an expanded dataset, more advanced machine learning techniques can be employed, such as deep neural networks or tree-based regression and classification algorithms.

## 5. Conclusions

Here, we introduce two novel algorithms that use both temporally and spatially resolved measurements and thereby improve the analytical performances of the MMB system. Compared with the Baseline approach, which uses only time-resolved measurements, the first algorithm (Feature 1) improves the detection performance (LoD) by a factor of 1.8 and the QR by ~25%. The second algorithm (Feature 2) improves the LoD by a factor of 2.4 and maintains the same QR as the Baseline approach. These results demonstrate the effectiveness of spatiotemporal processing.

The results also pave the way for advanced machine learning methods to extract and combine additional features to enhance the analytical performance even further. Because the algorithms are not correlated (each utilizes a different singular value), they can be combined, and thereby further improve the LoD and QR.

## Figures and Tables

**Table 1:** LoB and LoD of the Baseline approach and the proposed algorithms (Features 1 and 2).

Method	LoB [ng/L]	Gain	Gain (%)	LoD [ng/L]	Gain	Gain (%)
Baseline	0.170	-	-	0.384	-	-
Feature 1	0.082	2.1	(52%)	0.212	1.8	(44%)
Feature 2	0.042	4.1	(75%)	0.158	2.4	(58%)

**Table 2:** Quantitative resolutions (QRs) of the baseline approach and the proposed algorithms (Features 1 and 2). The gain is calculated as: $\left(Baseline\;QR-Feature\;QR\right)/Baseline\;QR$.

Concentration [ng/L]	Baseline QR [ng/L]	Feature 1 QR [ng/L]	Gain (%)	Feature 2 QR [ng/L]	Gain (%)
1	0.27	0.19	29.6%	0.24	11.0%
2	0.61	0.53	13.0%	0.64	−5.0%
5	1.57	1.17	25.4%	1.44	8.0%
20	6.73	4.99	25.8%	6.90	−2.5%

**Table 3:** *p*-values for Kolmogorov–Smirnov tests.

Concentration [ng/L]	Baseline	Feature 1	Feature 2
0	0.73	0.30	0.17
0.05	0.83	0.22	0.29
0.10	0.09	0.49	0.18
0.20	0.88	0.34	0.86
1.00	0.51	0.40	0.60

**Table 4:** *p*-values for Levene’s and Conover’s tests.

Test	Baseline	Feature 1	Feature 2
Levene	0.08	0.46	0.67
Conover	0.12	0.56	0.94

## Data Availability

The raw data supporting the conclusions of this article will be made available by the authors on request.
